# FSD-BRIEF: A Distorted BRIEF Descriptor for Fisheye Image Based on Spherical Perspective Model

**DOI:** 10.3390/s21051839

**Published:** 2021-03-06

**Authors:** Yutong Zhang, Jianmei Song, Yan Ding, Yating Yuan, Hua-Liang Wei

**Affiliations:** 1 Key Laboratory of Dynamics and Control of Flight Vehicle, Ministry of Education, School of Aerospace Engineering, Beijing Institute of Technology, Beijing 100081, China; 3120160041@bit.edu.cn (Y.Z.); sjm318@bit.edu.cn (J.S.); 2 The Department of Applied Mathematics, The University of Waterloo, Waterloo, ON N2L 3G1, Canada; yating.yuan@uwaterloo.ca; 3 Department of Automatic Control and Systems Engineering, University of Sheffield, Sheffield S1 3JD, UK; w.hualiang@sheffield.ac.uk

**Keywords:** fisheye camera, spherical perspective model, distorted BRIEF descriptor, feature point attitude matrix

## Abstract

Fisheye images with a far larger Field of View (FOV) have severe radial distortion, with the result that the associated image feature matching process cannot achieve the best performance if the traditional feature descriptors are used. To address this challenge, this paper reports a novel distorted Binary Robust Independent Elementary Feature (BRIEF) descriptor for fisheye images based on a spherical perspective model. Firstly, the 3D gray centroid of feature points is designed, and the position and direction of the feature points on the spherical image are described by a constructed feature point attitude matrix. Then, based on the attitude matrix of feature points, the coordinate mapping relationship between the BRIEF descriptor template and the fisheye image is established to realize the computation associated with the distorted BRIEF descriptor. Four experiments are provided to test and verify the invariance and matching performance of the proposed descriptor for a fisheye image. The experimental results show that the proposed descriptor works well for distortion invariance and can significantly improve the matching performance in fisheye images.

## 1. Introduction

For decades, feature detection and matching is one of the core areas of image processing in various applied fields, such as Visual based Simultaneously Localization and Mapping (V-SLAM), Structure from Motion (SfM), Augmented Reality (AR), general image retrieval, image mosaic, and image registration. Common features include Scale Invariant Feature Transform (SIFT) [1], Speed Up Robust Feature (SURF) [2], BRIEF [3] Oriented FAST and Rotated BRIEF (ORB) [4], KAZE [5], Binary Robust Invariant Scalable Keypoints (BRISK) [6], etc. and their derivations, such as Principle Component Analysis SIFT (PCA-SIFT) [7], Simplified-SIFT (SSIFT) [8], and Accelerated-KAZE (AKAZE) [9]. Neural network based features are also developed, such as L2-NET [10], HardNet [11], and AffNet [12]. These features are designed for pinhole images with little distortion and cannot achieve good performances for fisheye images with severe radial distortion.

Compared with a pinhole camera, a fisheye camera has a wide field of view (FoV), and the captured image contains more abundant information. This makes the fisheye camera extensively adopted in robot navigation, visual monitoring, virtual reality, visual measurement, and 3D reconstruction. However, due to the severe radial distortion of the fisheye image, adopting the common feature descriptors directly may lead to a significant reduction in matching performance.

In order to reduce the impact of distortion on the feature matching performance, we propose a novel distorted BRIEF descriptor based on the spherical perspective model, named Fisheye Spherical Distorted BRIEF (FSD-BRIEF). Firstly, we propose a method based on 3D gray centroid to determine the direction of each feature point in the spherical image. By constructing an attitude matrix of a feature point, the position and direction of the feature point in the spherical image can be described in a nonsingular form. In order to reduce the calculation error of the 3D gray centroid caused by uneven distribution of pixels in the spherical image, a pixel density function is designed to represent the degree of pixel density on the spherical surface by the size of the patch area mapped by each pixel in the fisheye image. We build an attitude coordinate system of each feature point and propose a coordinate mapping method to project the BRIEF descriptor template on the fisheye image. The distortion form of the projected BRIEF template is consistent with the image distortion near the feature point, which prevents the calculated BRIEF descriptor from the affection of the radial distortion in fisheye image. The main contributions of the paper include:

1. A new pixel density function represented by the area of the spherical surface patch that each pixel of fisheye image occupies;
2. A new method of determining the 3D gray centroid and the direction of feature points with pixel density function based on a spherical perspective model;
3. A new feature point attitude matrix, providing a nonsingular description for both the position and the direction of the feature point in the spherical image surface;
4. A novel descriptor template distortion method based on the spherical perspective model and the feature point attitude matrix.

The remaining of the paper is arranged as follows. In Section 2, the related work of the fisheye image point feature is presented. In Section 3, the notation of the perspective model is briefly introduced. Section 4 is about the method of determining and expressing the direction of feature point. Then the method of calculating the FSD-BRIEF descriptor is described. In Section 5, experimental results are provided and the performance of the proposed FSD-BRIEF is tested and verified. Section 6 briefly summarizes the work. In Section 7, the future work is stated.

## 2. Related Work

By virtue of its front lens protruding in a parabola shape, fisheye camera has a large FoV whose angle of view is close to or even more than 180°. Although this characteristic can maximize the angle of view, it brings severe radial distortion in its captured image, leading to different scale factors for pixels in different positions of the image. Thus, it could make the traditional feature descriptors designed for plane image fail to match in raw fisheye images [13,14].

Generally, the methods to extract descriptors in fisheye images can be divided into two main streams according to whether images are corrected or not: resampling and non-resampling approaches.

Resampling approaches [15,16,17] segment the FoV image into several sub-FoVs and correct them based on a plane perspective model, then feature descriptors can be extracted and matched on the corrected sub-FoV. Lin et al. [15] adopted a visual-inertial based UAV (Unmanned Aerial Vehicle) navigation system, where two sub-regions are sampled in the horizontal direction of the fisheye FoV to obtain two undistorted pinhole image fields, which cover 180° horizontal FoV, but they discarded the upper and lower parts of the vertical FoV. Miiller et al. [16] presented a robust visual inertial odometry and time-efficient omni-directional 3D mapping system, where the FoV of each fisheye camera is divided into two piecewise pinhole fields so as to overcome the distortion. However, some parts near the edge of the FoV are wasted. Wang et al. [17] proposed a new real-time feature-based simultaneous localization and mapping system, where a fisheye image is projected onto five surfaces of a cube, and then descriptors are extracted on the unfolded surfaces of the cube. However, the stretching distortion and seam distortion exist between surfaces, for example, a straight line will become a broken line. Thus, in the resampling approaches, the whole FoV of the fisheye image is hard to be fully utilized, and the continuity between sub-FoV cannot be guaranteed. In addition, due to the view geometry of the plane perspective model, there is a small stretching distortion in the edge of the sub-FoV.

Unlike the resampling approaches, which directly correct fisheye images to pinhole images, a non-resampling approach uses descriptors to describe features in fisheye images. For example, inspired by the planar SIFT framework [18,19,20], Arican et al. [21] designed a new scale invariant omni-directional SIFT feature based on Riemannian geometry. Lourenco et al. [22] proposed a Spherical Radial Distortion SIFT (sRD-SIFT) feature, where the extraction of the feature and the calculation of the descriptor was designed based on the spherical perspective model and the raw fisheye image without resampling. However, the improved algorithms based on SIFT are generally long time-consuming. Cruz-Mota et al. [23] and Hansen et al. [24] utilized spherical harmonic function as the basic function to study the spectral analysis of spherical panoramic images. Since Gaussian filtering on the sphere can be realized as a diffusion process through the spherical Fourier transform, spherical harmonic function is used to construct scale space on the sphere. In theory, the spherical harmonic function can be used to maintain the invariance of the descriptors to encounter the changes of the camera poses and positions. However, the spherical harmonic function usually needs a large amount of computation and has inherent bandwidth limitation. This greatly weakens the capability of dealing with large-scale matching problems and cannot meet the real-time requirements of many applications.

For improving the calculation speed, Qiang et al. [25] proposed Spherical ORB (SPHORB), a binary spherical feature based on the ORB feature, which is the first binary descriptor for a panoramic image based on hexagon geodesic grid. In essence, SPHORB is still a special resampling approach, which divides the spherical panoramic image into 20 regular triangle fields according to the shape of a regular icosahedron, and aligns the pixel of adjacent regular triangles seamlessly. However, in the hexagon geodesic grid, the image patches near the 12 vertices of the regular icosahedron are discarded due to the distortion of the pixel distribution pattern, resulting in 12 FoV holes occupying 1.4% of the total FoV.

Note that it can result in holes when resampling the fisheye image based on hexagon geodesic grid. To avoid this, Urban et al. [26] proposed a new distorted descriptor, called Masked Distorted BRIEF (mdBRIEF). Although this work distorts the descriptors to adapt to different image regions instead of correcting the distortion of the fisheye image, the direction angle of feature points is obtained in the raw fisheye image by calculating the gray centroid in a circle template, which is still affected by the fisheye image distortion. Furthermore, the descriptors are distorted excessively near the edge of the fisheye image since it is distorted based on the plane perspective model.

Most recently, Pourian et al. [27] proposed an end-to-end framework to enhance the precision of the descriptor matching between multiple wide-angle images. In their work, the global matching and the local matching of descriptors are combined in three stages. However, a new distortion in the edge of the corrected image is introduced when an equal rectangle image transformation is employed in the global matching stage, lowering the performance of the framework.

In summary, the binary descriptor that can make use of the whole FoV and keep invariance in each position of the fisheye image has not been proposed. In order to avoid the FoV holes caused by the resampling approaches, and reduce the excessive distortion of descriptors in large FoV images, in this paper, we design a novel Fisheye Spherical Distorted BRIEF (FSD-BRIEF) descriptor, which is a distorted binary feature descriptor based on the spherical perspective model for fisheye images.

## 3. Fisheye Camera Model

**Figure 1:**
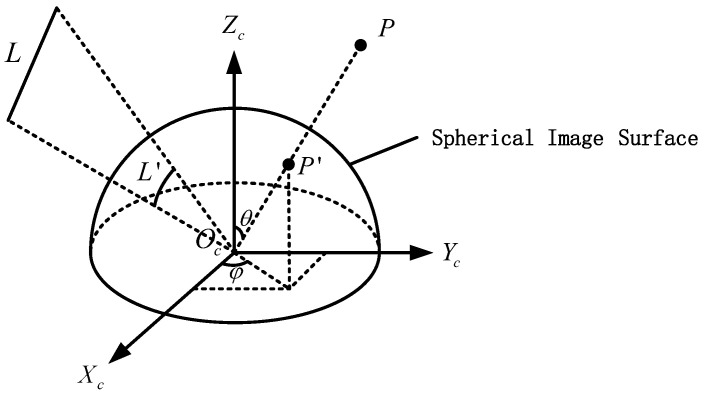
Spherical perspective model (θ: The FoV latitude angle; φ: the FoV longitude angle).

In this paper, in order to ensure that the FoV of the fisheye image can be fully utilized without losing the performance of the feature descriptor, a new descriptor FSD-BRIEF is designed based on spherical perspective model. Different from the plane perspective model, the projection surface is a unit sphere with the origin of camera coordinate system as center, so as to ensure that the scale factors of each position on the projection surface are consistent. The spherical perspective model and its perspective projection relationship are shown in Figure 1. We define the camera coordinate system as $O_{c}X_{c}Y_{c}Z_{c}$. The origin point $O_{c}$ is located at the optical center of the camera, the X-axis $O_{c}X_{c}$ points to the right along the long side of the imaging target surface, the Y-axis $O_{c}Y_{c}$ points downward along the wide edge direction of the imaging target surface, and the Z-axis $O_{c}Z_{c}$ points to the front of the camera along the optical axis direction. $P^{\prime }$ is the projection point on the spherical image surface of the space point *P*. $L^{\prime }$ is the projection large arc on the spherical image surface of the space Line *L*.

For a point *P* in a three-dimensional space, define its space coordinate in camera coordinate system as:

(1) $$P_{c}={\left[\begin{array}{ccc} x & y & z \end{array}\right]}^{T}$$

The projection point of *P* in the fisheye image is p, and its pixel coordinates are expressed as follows:

(2) $$p={\left[\begin{array}{cc} u & v \end{array}\right]}^{T}$$

In this paper, Kannala-Brandt4 (KB4) [28] model is used as the fisheye camera model, its mathematical form is shown below:

(3) $$\begin{array}{c} \theta =\arctan2(\sqrt{x^{2}+y^{2}},z)\\ \varphi =\arctan2(y,x)\\ \theta_{d}=\theta \left(1+k_{1}\theta^{2}+k_{2}\theta^{4}+k_{3}\theta^{6}+k_{4}\theta^{8}\right)\\ u=f_{x}\theta_{d}\cos\varphi +c_{x}\\ v=f_{y}\theta_{d}\sin\varphi +c_{y} \end{array}$$

where $f_{x}$ and $f_{y}$ are the horizontal and vertical focal length of the camera, $c_{x}$ and $c_{y}$ are the coordinates of the principal points of the camera, and $k_{1}$, $k_{2}$, $k_{3}$, $k_{4}$ are the distortion coefficients. θ is the FoV latitude angle, which represents the angle between the $O_{c}Z_{c}$ axis and the vector $\vec{O_{c}P}$. φ is the FoV longitude angle, which denotes the angle between the $O_{c}X_{c}$ axis and the projection vector of $\vec{O_{c}P}$ on the $X_{c}O_{c}Y_{c}$ plane. $\theta_{d}$ is the angle θ as deflected by the fisheye lens. The arctan2 is the quadrant aware version of arctangent function.

Based on the spherical perspective model in Equation (3), Π represents the mapping function. The mapping from the point $P_{c}$ to the pixel point p in fisheye image can be expressed as:

(4) $$p=\Pi (P_{c})$$

The inverse mapping function of Π is defined as $\Pi^{-1}$, which indicates the mapping from the point p to the point $P^{\prime }$ on the spherical image surface as follows:

(5) $${P_{c}}^{\prime }=\Pi^{-1}\left(p\right)$$

where $P_{c}^{\prime }$ is the coordinate vector of point $P^{\prime }$ in the camera coordinate system. Notice that $\left|{P_{c}}^{\prime }\right|=\sqrt{x^{2}+y^{2}+z^{2}}=1$.

## 4. FSD-BRIEF Descriptor

The procedure of extracting the FSD-BRIEF descriptor includes four steps, namely, pixel density function designing, 3D gray centroid calculation, feature point attitude matrix construction, and FSD-BRIEF descriptor extraction. In the spherical perspective model, the densities of pixels are distributed unevenly, lowering the effectiveness of descriptors. Thus, a pixel density function is proposed firstly to calculate the distribution compensation of each pixel so as to reduce the effect of uneven pixel distribution. Then, with the help of the pixel density function, a more accurate 3D gray centroid is designed to determine the direction of FSD-BRIEF descriptor and keep its rotation invariance in the spherical perspective model. Next, we further devise a nonsingular form, a feature point attitude matrix, to represent the position and the direction of a feature point. Finally, based on the feature point attitude matrix, an FSD-BRIEF descriptor is extracted by a constructed coordinate mapping relation between the BRIEF template and the raw fisheye image.

### 4.1. Pixel Density Function Designing

In this section, by defining the pixel density function, the distribution density of pixels on the unit sphere surface is expressed numerically.

Assuming that a pixel p in a fisheye image occupies a small patch PIX_PATCH(p) of the corresponding unit sphere, the mathematical expression of PIX_PATCH(p) is given by:

(6) $$PIX\_PATCH\left(p\right)=\left\{\left.\Pi^{-1}\left(p+\Delta p\right)\right|\Delta p={\left[\begin{array}{cc} \Delta u & \Delta v \end{array}\right]}^{T},-\frac{1}{2}<\Delta u<\frac{1}{2},-\frac{1}{2}<\Delta v<\frac{1}{2}\right\}$$

where Δu and Δv are the coordinate offsets under the pixel coordinate system in the fisheye image. It is obvious that the area of the patch PIX_PATCH(p) will be smaller if the distance between point p and its adjacent pixels is closer, which means that the pixel density of point p is denser.

Therefore, the pixel density function m(p) is defined as the area of the patch PIX_PATCH(p). To simplify the computation of the curved surface area, we assume that the patch size is small enough to approximate as a parallelogram, so the pixel density function compensation m(p) can be computed by:

(7) $$m\left(p\right)=\left\{\begin{array}{c} \frac{1}{4}{∥\left[\Pi^{-1}\left(p+\Delta x\right)-\Pi^{-1}\left(p-\Delta x\right)\right]\times \left[\Pi^{-1}\left(p+\Delta y\right)-\Pi^{-1}\left(p-\Delta y\right)\right]∥}_{2},p\in I\\ 0,p\notin I \end{array}\right.$$

where ${∥▪∥}_{2}$ means L2 norm operation, and Δx,Δy are the coordinate offsets as follows:

(8) $$\begin{array}{c} \Delta x={\left[\begin{array}{cc} 1 & 0 \end{array}\right]}^{T}\\ \Delta y={\left[\begin{array}{cc} 0 & 1 \end{array}\right]}^{T} \end{array}$$

From Equation (7), the pixel density function m(p) of the whole FoV only depends on the mapping function Π of the spherical perspective model in Equation (4).

### 4.2. 3D Gray Centroid Calculation

**Figure 2:**
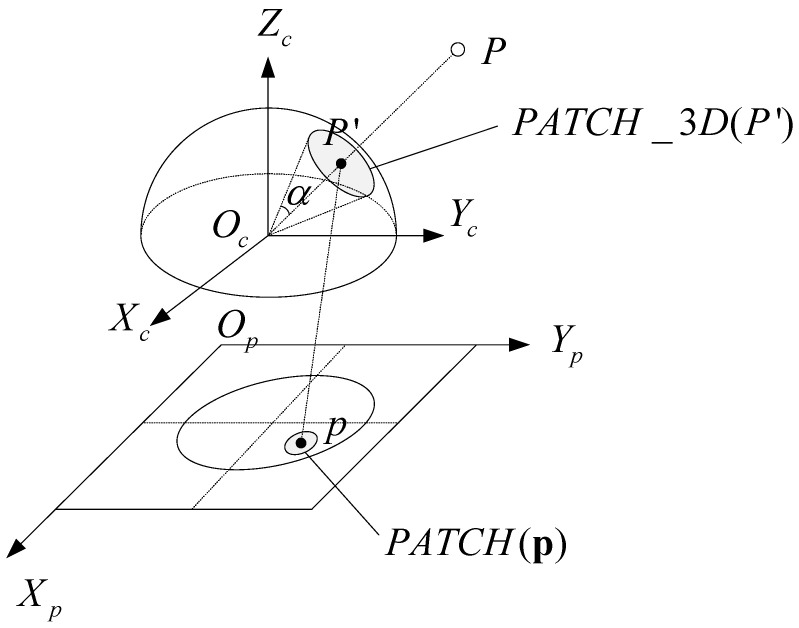
The circle area for 3D gray centroid calculation on the unit spherical surface and its projection area in the fisheye image plane.

To determine the direction of the FSD-BRIEF descriptor, we propose a 3D gray centroid. Compared with 2D gray centroid [13,14,26], the proposed 3D gray centroid is more accurate since it takes full advantage of the consistent scale factor on the spherical perspective model. The 3D gray centroid is calculated in a circle area on the unit spherical surface. Figure 2 illustrates the correspondence of the circle area between the unit spherical surface and the fisheye image plane. As shown in Figure 2, for a FAST (Features From Accelerated Segment Test) [29] feature point p, its projection point on the unit spherical surface is $P^{\prime }$, and its 3D gray centroid calculation area is the circle area $PATCH\_3D(P^{\prime })$ with $P^{\prime }$ as the center. PATCH(p) is the projection of the $PATCH\_3D(P^{\prime })$ in the fisheye image plane $O_{P}X_{P}Y_{P}$. α is half of the apex angle of the cone formed by $PATCH\_3D(P^{\prime })$ and the origin point $O_{c}$.

Note that the horizontal and vertical angular resolutions of fisheye cameras are approximately $f_{x}$ and $f_{y}$ (Pixels Per Radian) in KB4 model, and the values of $f_{x}$ and $f_{y}$ are often very close. In order to make the radius of the circular range cover about 15 pixel width while ensure the same mathematical status of $f_{x}$ and $f_{y}$, the value of α in radians is selected as 15 divided by the arithmetic mean of $f_{x}$ and $f_{y}$, that is,

(9) $$\alpha =\frac{15}{\frac{f_{x}+f_{y}}{2}}=\frac{30}{f_{x}+f_{y}}$$

Define the projection area PATCH(p) as:

(10) $$PATCH\left(p\right)=\left\{\left.\left(p+\Delta p\right)\right|\Pi^{-1}\left(p+\Delta p\right)\cdot \Pi^{-1}\left(p\right)>\cos\alpha \right\}$$

where Δp is the offset from the pixel p to the pixel in the area PATCH(p) in the fisheye image plane. $\Pi^{-1}\left(p\right)$ is the position vector of $P^{\prime }$. $P^{\prime }$ is also the projection point of the pixel p on the unit sphere. $\Pi^{-1}\left(p+\Delta p\right)$ represents the position vector of the projection point of the pixel p+Δp on the unit sphere. $\Pi^{-1}\left(p+\Delta p\right)\cdot \Pi^{-1}\left(p\right)>\cos\alpha$ means that the angle between the two vectors $\Pi^{-1}\left(p+\Delta p\right)$ and $\Pi^{-1}\left(p\right)$ is less than α. The region PATCH(p) is actually the projection area of the region $PATCH\_3D(P^{\prime })$ on the fisheye image.

The 3D gray centroid of the feature point p is defined as *C*. The symbol $C_{c}$ denotes the coordinate vector of *C* in the camera coordinate system. The calculation formula of $C_{c}$ is:

(11) $$C_{c}=\frac{\sum_{\overset{⌢}{p}\in PATCH\left(p\right)}\Pi^{-1}\left(\overset{⌢}{p}\right)m\left(\overset{⌢}{p}\right)I\left(\overset{⌢}{p}\right)}{\sum_{\overset{⌢}{p}\in PATCH\left(p\right)}m\left(\overset{⌢}{p}\right)I\left(\overset{⌢}{p}\right)}$$

where $\overset{⌢}{p}$ is a pixel in PATCH(p). $I(p_{k})$ represents the gray value of the pixel $\overset{⌢}{p}$ in PATCH(p), $m(\overset{⌢}{p})$ is the pixel density function value of $\overset{⌢}{p}$, $\Pi^{-1}\left(\overset{⌢}{p}\right)$ indicates the 3D coordinate of the projection point on the unit sphere surface of $\overset{⌢}{p}$.

### 4.3. Feature Point Attitude Matrix Construction

**Figure 3:**
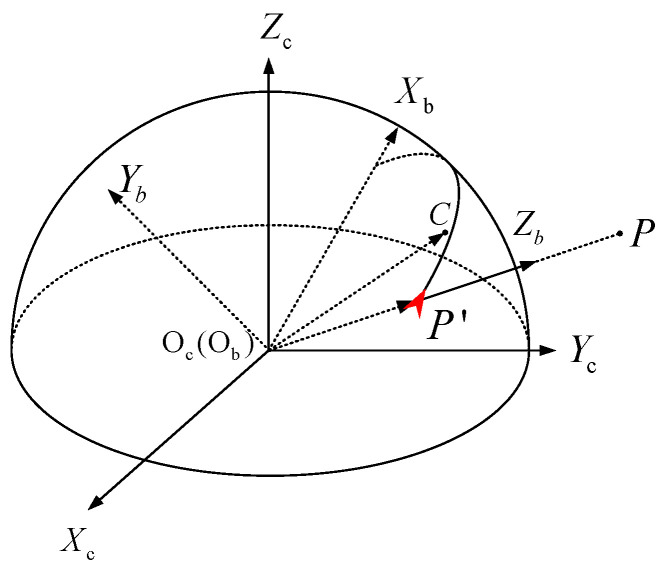
Feature point attitude coordinate system.

In order to avoid the singularity of direction expression of feature points on the poles of the unit spherical surface [25], we propose a feature point attitude matrix, a nonsingular expression, to represent the position and the direction of a feature point. The feature point attitude coordinate system $O_{b}X_{b}Y_{b}Z_{b}$ is shown in Figure 3. The origin point $O_{b}$ coincides with the origin point $O_{c}$ of the camera coordinate system. The Z-axis $O_{b}Z_{b}$ coincides with the vector $P_{c}^{\prime }$. The Y-axis $O_{b}Y_{b}$ is consistent with the $P_{c}^{\prime }\times C_{c}$. The X-axis $O_{b}X_{b}$ direction is determined by right-hand rule. The X-axis is coplanar with the 3D gray centroid vector $C_{c}$ and the position vector $P_{c}^{\prime }$.

The coordinate transformation matrix $R_{cb}$ from the feature point attitude coordinate system to the camera coordinate system can be obtained as follows:

(12) $$R_{cb}=\left[\begin{array}{ccc} \frac{C_{c}-\frac{C_{c}\cdot {P_{c}}^{\prime }}{P_{c}{{}^{\prime }}^{2}}{P_{c}}^{\prime }}{\left|C_{c}-\frac{C_{c}\cdot {P_{c}}^{\prime }}{P_{c}{{}^{\prime }}^{2}}{P_{c}}^{\prime }\right|} & \frac{{P_{c}}^{\prime }\times C_{c}}{\left|{P_{c}}^{\prime }\times C_{c}\right|} & {P_{c}}^{\prime } \end{array}\right]$$

The matrix $R_{cb}$ is defined as feature point attitude matrix.

### 4.4. FSD-BRIEF Descriptor Extraction

In this section, to enhance the distortion invariance of the descriptor in the fisheye image, FSD-BRIEF will be extracted by distorting the BRIEF template based on the constructed feature point attitude matrix so that its template can fit the distortion form of the adjacent area of the feature point.

**Figure 4:**
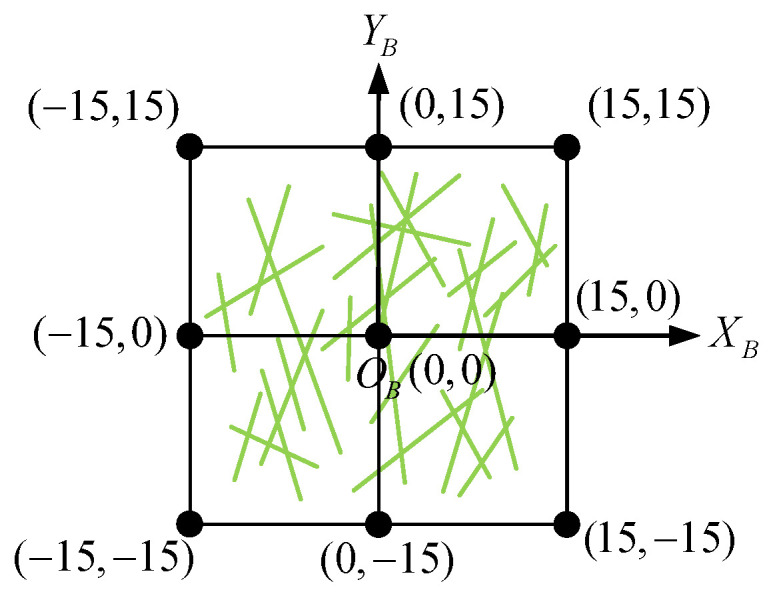
BRIEF template and its coordinate system.

At first, for a feature point, we define its square neighborhood region as a BRIEF template with a coordinate system $O_{B}X_{B}Y_{B}$ whose origin point $O_{B}$ is located at the feature point and coordinate ranges from −15 to 15, as shown in Figure 4. The green lines are the selected 256 groups of pixel pairs on the template.

**Figure 5:**
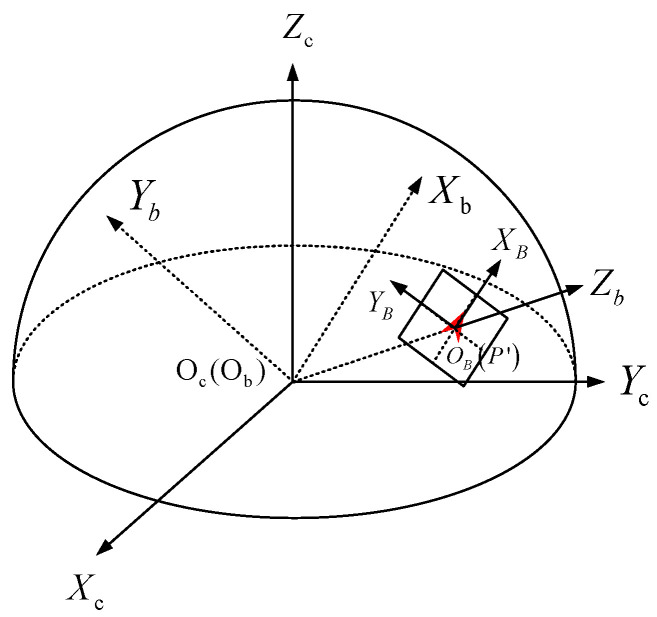
Position relationship between BRIEF template and spherical projection surface.

Then, the defined BRIEF template is scaled to a certain extent and placed at the feature point as shown in Figure 5. For doing so, the following three conditions must be satisfied:

1. The center point $O_{B}$ of the descriptor template coincides with the projection point $P^{\prime }$ of the feature point p on the sphere. In other words, the coordinate of point $O_{B}$ in the feature point attitude coordinate system is ${\left[\begin{array}{ccc} 0 & 0 & 1 \end{array}\right]}^{T}$.
2. The directions of $O_{B}X_{B}$, $O_{B}Y_{B}$ axis of BRIEF template coordinate system are consistent with the directions of $O_{b}X_{b}$, $O_{b}Y_{b}$ axis of the feature point attitude coordinate system.
3. There is a scale factor $\frac{\alpha }{15}$ between the coordinates in the BRIEF template coordinate system and the coordinates in the feature point attitude coordinate system.

**Figure 6:**
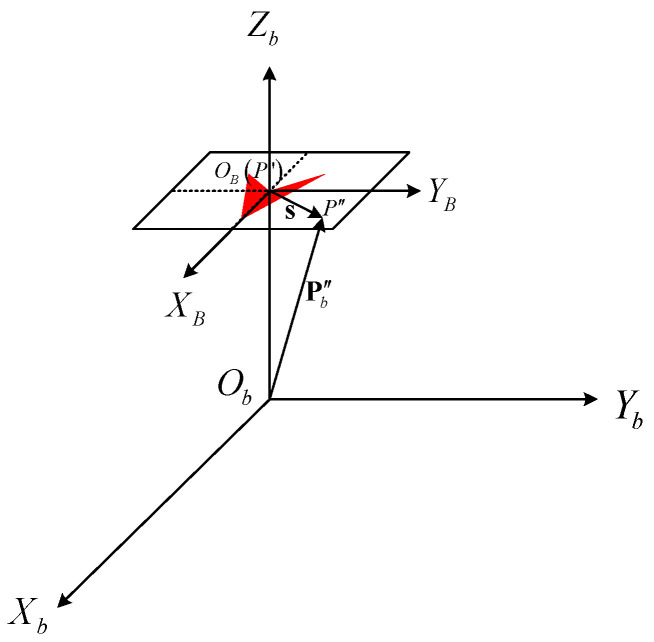
Coordinate mapping between BRIEF template coordinate system and feature point attitude coordinate system.

Figure 6 shows a zoom-in of a local area along the direction of $O_{b}Z_{b}$ in Figure 5 at the feature point $P^{\prime }$. As shown in Figure 6, for a point $P^{\prime \prime }$ on the BRIEF template, its homogeneous coordinate vector in $O_{B}X_{B}Y_{B}$ coordinate system is s. The coordinate vector of point $P^{\prime \prime }$ in the feature point attitude coordinate system is $P_{b}^{\prime \prime }$. Then, the $P_{b}^{\prime \prime }$ can be solved by:

(13) $$P_{b}^{\prime \prime }=\mathrm{Ds}$$

where

(14) $$\begin{array}{c} D=diag(\frac{\alpha }{15},\frac{\alpha }{15},1)\\ s={\left[\begin{array}{ccc} s_{x} & s_{y} & 1 \end{array}\right]}^{T} \end{array}$$

According to the law of 3D coordinate transformation and the $P_{b}^{\prime \prime }$, the coordinate $P_{c}^{\prime \prime }$ of point $P^{\prime \prime }$ in the camera coordinate system can be calculated by:

(15) $$P_{c}^{\prime \prime }=R_{cb}P_{b}^{\prime \prime }$$

where $R_{cb}$ is the feature point attitude matrix.

The projection point $p^{\prime \prime }$ of $P_{c}^{\prime \prime }$ in the fisheye image can be obtained by:

(16) $$p^{\prime \prime }=\Pi \left(P_{c}^{\prime \prime }\right)$$

To sum up, for a feature point whose attitude matrix is $R_{cb}$, the coordinate mapping relationship between the point s in the BRIEF template and the projection point $p^{\prime \prime }$ in the fisheye image is:

(17) $$p^{\prime \prime }=\Pi \left(R_{cb}\mathrm{Ds}\right)$$

**Figure 7:**
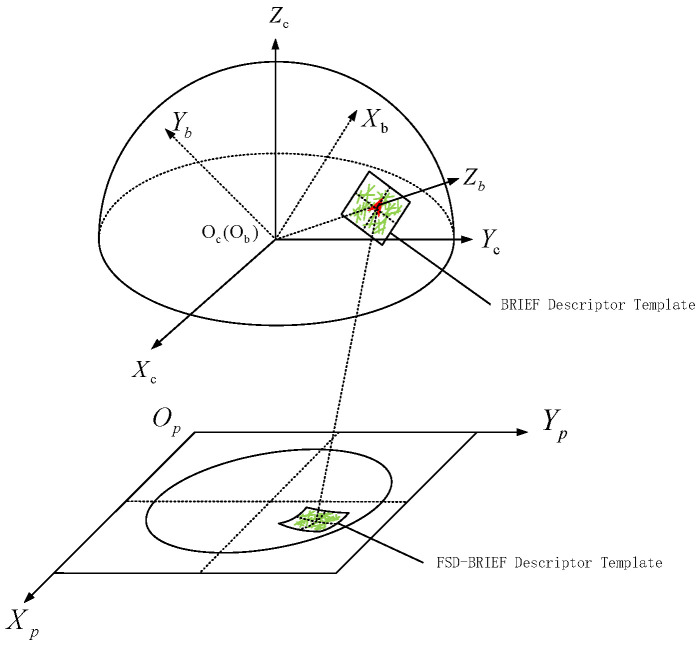
General view of FSD-BRIEF descriptor.

According to Equation (17), the FSD-BRIEF of a feature point can be extracted by the calculated projection points of the FSD-BRIEF template in the fisheye image. Figure 7 shows the general view of the FSD-BRIEF descriptor. It is clear that the FSD-BRIEF template in the fisheye image changes with the position where the feature point is located, so as to ensure that the descriptor is adaptive to the different distortions in the fisheye image, and achieves a good performance on distortion invariance.

## 5. Experimental Evaluation

In this section, we present four experiments that were used for evaluating the performance of the proposed method. Experiment 1 was an ablation experiment carried out on a virtual dataset, which was used to verify the contribution of pixel density function towards improving the solution accuracy of FSD-BRIEF orientation. Experiment 2 was also conducted on the virtual dataset, aiming to prove the invariance of FSD-BRIEF compared with three BRIEF-based descriptors. Experiment 3 and Experiment 4 were performed to evaluate the matching performance of FSD-BRIEF under (1) different camera motions on a real dataset, and (2) distortion conditions on sRD-SIFT dataset [22], respectively. The results of these two experiments were compared with those produced by five state-of-the-art features.

### 5.1. Experiment 1: The Contribution Evaluation of the Pixel Density Function to the Accuracy of Feature Point Orientation

**Dataset:** In this experiment, we investigated the contribution of the pixel density function to the accuracy of feature point orientation. In order to have accurate ground truth of the direction of feature points, we produced a virtual dataset by simulating a projection of the first image of the Graffiti dataset [30]; this was used as a test image to two virtual fisheye cameras with different intrinsic parameters. At first, in the test image, $N_{p}$ feature points $p_{t}^{i}\left(i=1,2,\ldots ,N_{p}\right)$ were extracted. During the generation of the virtual dataset, the test image and a selected virtual fisheye camera were placed in the same virtual space. By placing the test image in different poses, we projected each feature point in the fisheye image on several selected positions with different longitude angle φ and latitude angle θ. The relationship between the angle φ, θ and the pose of the test image is shown in Appendix A. φ takes $N_{\varphi }$ values and θ takes $N_{\theta }$ values. For each virtual fisheye camera, $N_{p}\times N_{\varphi }\times N_{\theta }$ test samples were generated. Each test sample consisted of a generated fisheye image $I(\varphi ,\theta ,p_{t}^{i})$, a corresponding feature point position $p_{c}^{i}\left(\varphi ,\theta ,p_{t}^{i}\right)$ in the fisheye image, and a ground truth feature point attitude matrix ${R_{cb}^{i}}^{*}\left(\varphi ,\theta ,p_{t}^{i}\right)$. More details of the dataset are given in Appendix B.

**Baseline:** To verify the effectiveness of the pixel density function compensation proposed in this paper, we compared two algorithms, namely, the feature point attitude matrix computation part of FSD-BRIEF without the compensation (version 1) and with (version 2). In version 1, the 3D gray centroid was calculated without the pixel density compensation term $m(\overset{⌢}{p})$. That is, the gray centroid computation formula of version 1 is shown as Equation (18). In version 2, we used Equation (11) to calculate the 3D gray centroids of feature points.

(18) $$C_{c}=\frac{\sum_{\overset{⌢}{p}\in PATCH\left(p\right)}\Pi^{-1}\left(\overset{⌢}{p}\right)I\left(\overset{⌢}{p}\right)}{\sum_{\overset{⌢}{p}\in PATCH\left(p\right)}I(\overset{⌢}{p})}$$

**Fisheye cameras:** In order to verify the contribution of the pixel density function under different FoV cameras, two virtual cameras with different FoVs were selected for this experiment. Table 1 shows the intrinsic parameters of the two cameras.

**Figure 8:**
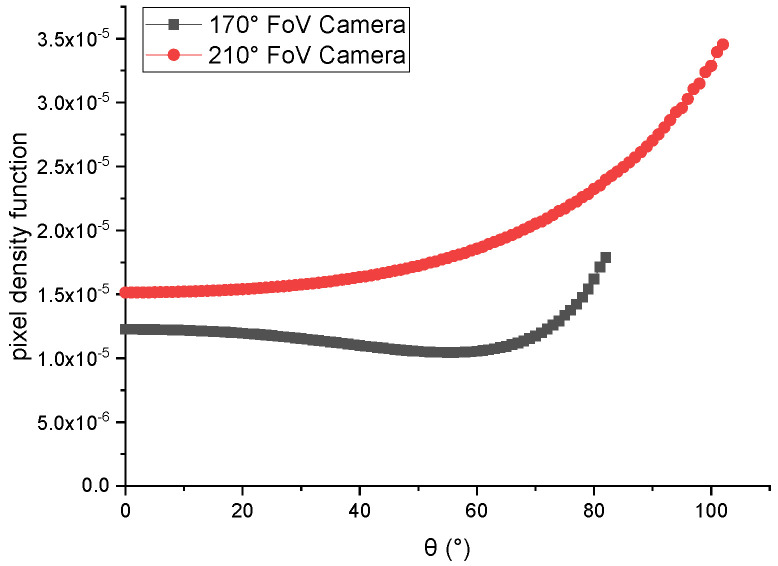
The pixel density function of 170° field of view (FoV) and 210° FoV camera with θ.

Figure 8 shows the curve of the pixel density function of 170° FoV and 210° FoV cameras with θ. From the curve, we can see that the curve of the pixel density function of 170° FoV cameras decreased in angle range 0–60°, and increased in angle range 60–80°. Another curve, which was for the pixel density function of 210° FoV camera, increased in the whole angle range of 0–90°.

**Figure 9:**
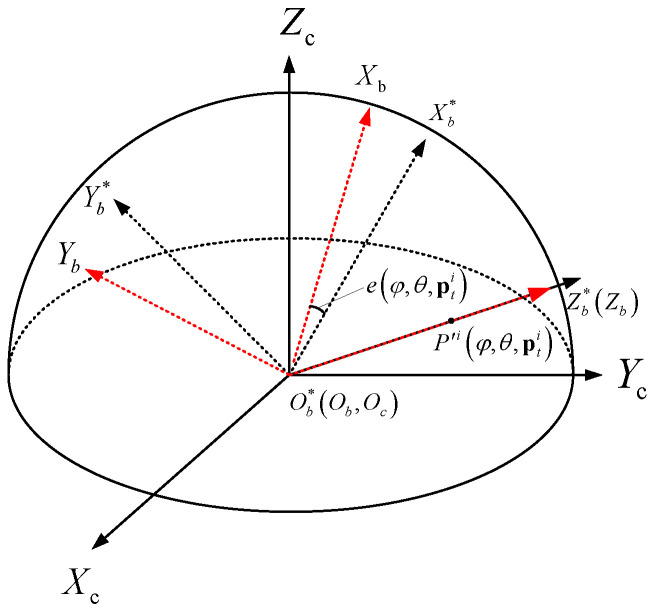
The definition of feature point direction angle error between calculated direction and ground truth direction.

**Evaluation metrics:** In the experimental verification process, the direction angle error of the feature point is used for quantitative evaluation. The direction angle error, denoted by $e(\varphi ,\theta ,p_{t}^{i})$, is shown in Figure 9, where $P^{i}\left(\varphi ,\theta ,p_{t}^{i}\right)$ is the projection point of $p_{c}^{i}\left(\varphi ,\theta ,p_{t}^{i}\right)$ on the unit sphere surface. The coordinate system $O_{b}^{*}X_{b}^{*}Y_{b}^{*}Z_{b}^{*}$ is the feature point attitude coordinate system corresponding to the ground truth feature point attitude matrix ${R_{cb}^{i}}^{*}\left(\varphi ,\theta ,p_{t}^{i}\right)$, whilst $O_{b}X_{b}Y_{b}Z_{b}$ is the feature point attitude coordinate system corresponding to the calculated feature point attitude matrix $R_{cb}^{i}\left(\varphi ,\theta ,p_{t}^{i}\right)$. Note that $\vec{O_{b}^{*}X_{b}^{*}}$ and $\vec{O_{b}X_{b}}$ are defined as the ground truth direction and the calculated direction of the feature point (see Section 4.3). The unit of $e(\varphi ,\theta ,p_{t}^{i})$ is defined as degree (°). Let ${\left(\vec{O_{b}^{*}X_{b}^{*}}\right)}_{c}$ and ${\left(\vec{O_{b}X_{b}}\right)}_{c}$ be the coordinate of the unit direction vectors corresponding to $\vec{O_{b}^{*}X_{b}^{*}}$ and $\vec{O_{b}X_{b}}$ in the camera coordinate system, then:

(19) $$\begin{array}{c} {\left(\vec{O_{b}^{*}X_{b}^{*}}\right)}_{c}={R_{cb}^{i}}^{*}\left(\varphi ,\theta ,p_{t}^{i}\right){\left[\begin{array}{ccc} 1 & 0 & 0 \end{array}\right]}^{T}\\ {\left(\vec{O_{b}X_{b}}\right)}_{c}=R_{cb}^{i}\left(\varphi ,\theta ,p_{t}^{i}\right){\left[\begin{array}{ccc} 1 & 0 & 0 \end{array}\right]}^{T}\\ \cos\left(\frac{\pi }{180}e\left(\varphi ,\theta ,p_{t}^{i}\right)\right)=\vec{O_{b}X_{b}}\cdot \vec{O_{b}^{*}X_{b}^{*}}={\left(\vec{O_{b}X_{b}}\right)}_{c}^{T}{\left(\vec{O_{b}^{*}X_{b}^{*}}\right)}_{c} \end{array}$$

From Equation (19), we can obtain the expression of $e(\varphi ,\theta ,p_{t}^{i})$ as:

(20) $$e\left(\varphi ,\theta ,p_{t}^{i}\right)=\frac{180}{\pi }\arccos\left(\left[\begin{array}{ccc} 1 & 0 & 0 \end{array}\right]R_{cb}^{i}\left(\varphi ,\theta ,p_{t}^{i}\right){R_{cb}^{i}}^{*}\left(\varphi ,\theta ,p_{t}^{i}\right){\left[\begin{array}{ccc} 1 & 0 & 0 \end{array}\right]}^{T}\right)$$

Note that values of $e(\varphi ,\theta ,p_{t}^{i})$ could be calculated from experimental results indexed by φ (FoV longitude angle), θ (FoV Latitude Angle) and *i* (feature point index in test image). For an ideal method, $e(\varphi ,\theta ,p_{t}^{i})$ is always zero, and the calculated direction of feature point is consistent with the real direction. In fact, due to the influence of noise, the angle error $e(\varphi ,\theta ,p_{t}^{i})$ would not be zero. In this experiment, the smaller the value of $e(\varphi ,\theta ,p_{t}^{i})$, the more accurate the calculated feature point direction.

In this study, the mean error $e_{mean}\left(\theta \right)$ and the standard deviation $e_{SD}\left(\theta \right)$ were used to evaluate the results of $e\left(\varphi ,\theta ,p_{t}^{i}\right)$. $e_{mean}\left(\theta \right)$ measures the average error of the feature point direction calculated by using all the points under the latitude angle θ. $e_{SD}\left(\theta \right)$ measures the dispersion of the $e\left(\varphi ,\theta ,p_{t}^{i}\right)$ distribution under θ. $e_{mean}\left(\theta \right)$ and $e_{SD}\left(\theta \right)$ are calculated as follows:

(21) $$\begin{array}{c} e_{mean}\left(\theta \right)=\frac{\sum_{\varphi }\sum_{i}e(\varphi ,\theta ,p_{t}^{i})}{N_{\varphi }N_{p}}\\ e_{SD}\left(\theta \right)=\sqrt{\frac{\sum_{\varphi }{\sum_{i}\left[e\left(\varphi ,\theta ,p_{t}^{i}\right)-e_{mean}\left(\theta \right)\right]}^{2}}{N_{\varphi }N_{p}}} \end{array}$$

where, the $N_{\varphi }$ and $N_{p}$ are the number of φ and *i* values. The smaller the $e_{mean}\left(\theta \right)$ is, the more accurate the feature point direction is. The smaller the $e_{SD}\left(\theta \right)$ is, the more stable the result of feature point direction is.

**Figure 10:**
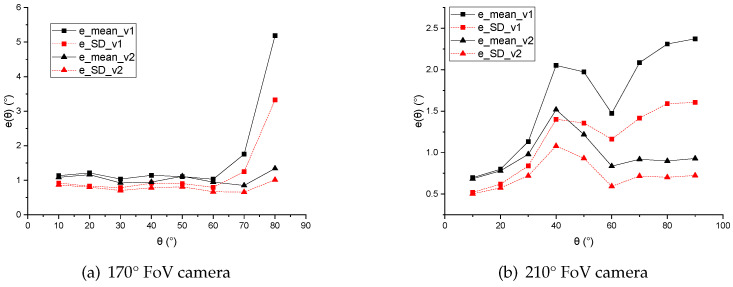
e−θ curves of two versions of feature point direction calculation methods in 170° FoV camera and 210° FoV camera.

**Evaluations:** In the 170° FoV camera, the range of θ is 10–80°. In the 210° FoV camera, the range of θ is 10–90°. The two statistics $e_{mean}\left(\theta \right)$ and $e_{SD}\left(\theta \right)$ are computed for both of the two cases. The comparison results are shown in Table 2 and Table 3. The error reduction of version 2 compared to version 1 are calculated as follows:

(22) $$\eta =\frac{e_{v2}-e_{v1}}{e_{v1}}\times 100\%$$

where η is the value of error reduction, $e_{v1}$ and $e_{v2}$ are the value of the direction angle error of version 1 and version 2 individually. Taking the horizontal axis as the θ value and the vertical axis as $e_{mean}\left(\theta \right)$ and $e_{SD}\left(\theta \right)$, the e−θ curves are also drawn in Figure 10.

For the 170° FoV camera, both of the two compensation schemes led to similarly stable results in the angle range of 10–60°. However, when the angle θ became large (especially in the range of 60–80°), the performance of Version 2 was obviously much better than that of Version 1. Both of the average angle error and the accuracy dispersion of the proposed method (version 2) were about 1° in the whole fisheye FoV of the dataset.

For the 210° FoV camera, the overall performance of Version 2 was continuously better than that of Version 1 throughout the range of 30–90°.

The experimental results showed that near the edge of FoV, especially in the FoV region where the pixel density function increased monotonously with the angle θ, the pixel density compensation improved the accuracy and stability of feature point direction calculation significantly.

### 5.2. Experiment 2: Descriptor Invariance Evaluation of Fisheye Images in Different FoV Positions

**Baselines:** In this experiment, three typical BRIEF descriptors, including ORB, dBRIEF (Distorted BRIEF), and mdBRIEF, were selected as baselines. The descriptor of the feature point in each test sample in the virtual dataset generated in Experiment 1 was extracted by the tested features (FSD-BRIEF, ORB, dBRIEF, and mdBRIEF). In order to ensure a fair comparison of experimental results, all the binary descriptors were chosen to be 256 bits. dBRIEF is the version of mdBRIEF without on-line mask learning. For dBRIEF and mdBRIEF, we used the open source version provided in GitHub. For ORB, we used the functions provided in OpenCV and its default parameter settings.

**Evaluation metrics:** In this experiment, we define $D(\varphi ,\theta ,p_{t}^{i})$ as the descriptor of the feature point $p_{c}^{i}\left(\varphi ,\theta ,p_{t}^{i}\right)$. The associated Hamming distance error $\Delta D(\varphi ,\theta ,p_{t}^{i})$ of the descriptor of the feature point was used to evaluate the invariance performance of algorithms. $\Delta D(\varphi ,\theta ,p_{t}^{i})$ is calculated for each feature point test sample by each test feature as:

(23) $$\Delta D\left(\varphi ,\theta ,p_{t}^{i}\right)=h\left(D\left(\varphi ,\theta ,p_{t}^{i}\right),D\left(\varphi_{0},\theta_{0},p_{t}^{i}\right)\right)$$

here we selected $D(\varphi_{0},\theta_{0},p_{t}^{i})$ as the reference standard descriptor to compute the Hamming distance error, where $\varphi_{0}=45^{{}^{\circ}}\;\theta_{0}=10^{{}^{\circ}}$. For an ideal feature algorithm, for the same $p_{t}^{i}$, no matter what values of φ and θ take, there is $\Delta D(\varphi ,\theta ,p_{t}^{i})=0$. However, in practice, due to the resampling error of the fisheye camera, $\Delta D(\varphi ,\theta ,p_{t}^{i})$ was not zero. Therefore, the smaller the calculated value of $\Delta D(\varphi ,\theta ,p_{t}^{i})$, the stronger the invariance of the feature algorithm to radial distortion of the fisheye image.

Similar to Experiment 1, $\Delta D_{mean}\left(\theta \right)$ and $\Delta D_{SD}\left(\theta \right)$ were used as evaluation metrics. $\Delta D_{mean}\left(\theta \right)$ is the average value of the descriptor distance calculated by using all the points under the latitude angle θ. $\Delta D_{SD}\left(\theta \right)$ is the dispersion of the $\Delta D\left(\varphi ,\theta ,p_{t}^{i}\right)$ distribution under θ. The smaller the $\Delta D_{mean}\left(\theta \right)$ is, the stronger the invariance of feature algorithm to radial distortion of fisheye images. The smaller the $\Delta D_{SD}\left(\theta \right)$ is, the more stable the performance of the feature algorithm is. The computation formula of $\Delta D_{mean}\left(\theta \right)$ and $\Delta D_{SD}\left(\theta \right)$ was as follows:

(24) $$\begin{array}{c} \Delta D_{mean}\left(\theta \right)=\frac{\sum_{\varphi }\sum_{i}\Delta D(\varphi ,\theta ,p_{t}^{i})}{N_{\varphi }N_{p}}\\ \Delta D_{SD}\left(\theta \right)=\sqrt{\frac{\sum_{\varphi }{\sum_{i}\left[\Delta D\left(\varphi ,\theta ,p_{t}^{i}\right)-\Delta D_{mean}\left(\theta \right)\right]}^{2}}{N_{\varphi }N_{p}}} \end{array}$$

**Figure 11:**
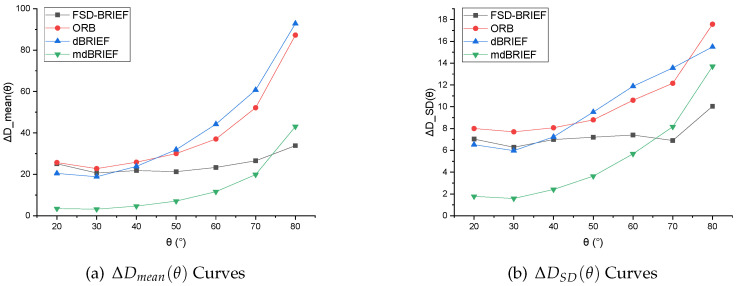
ΔD−θ curve results in 170° FoV camera.

**Figure 12:**
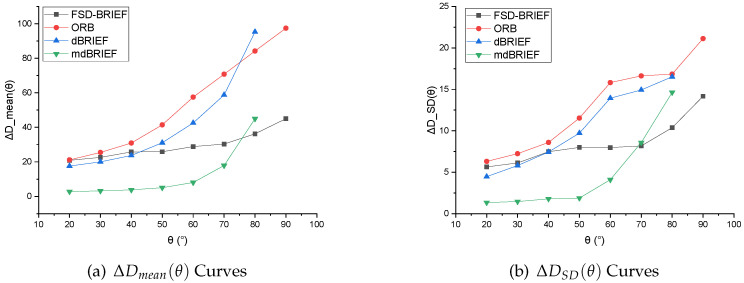
ΔD−θ curve results in 210° FoV camera.

**Evaluations:** Since $\theta_{0}=10^{{}^{\circ}}$ was set for the reference standard descriptor $D(\varphi_{0},\theta_{0},p_{t}^{i})$, the ranges of θ were selected as 20–80° in 170° FoV camera, and 20–90° in 210° FoV camera respectively. The values of $\Delta D_{mean}\left(\theta \right)$ and $\Delta D_{SD}\left(\theta \right)$ of FSD-BRIEF, ORB, dBRIEF, and mdBRIEF were computed. The numerical results are shown in Table 4 and Table 5. The corresponding curves of $\Delta D_{mean}\left(\theta \right)$ are shown in Figure 11, and the curves of $\Delta D_{SD}\left(\theta \right)$ are shown in Figure 12.

The experimental results of the two cameras showed that, in the angle range of 20–40°, FSD-BRIEF led to similarly stable descriptor errors as ORB and dBRIEF. However, in the angle range of 40–80°, the descriptor errors of ORB and dBRIEF tended to increase significantly, while the descriptor errors of FSD-BRIEF increased much less than that of ORB and dBRIEF. In the angle range of 75–80°, the descriptor error of FSD-BRIEF was smaller than that of mdBRIEF. However, the descriptor error of FSD-BRIEF was larger than that of mdBRIEF in the angle range of 20–60°; this is because an on-line mask learning scheme was performed in mdBRIEF, where the unstable binary bits were masked.

The standard deviations (SD) of FSD-BRIEF, ORB and dBRIEF were similar in the angle range of 20–40°. In the angle range up to 50°, the SD of FSD-BRIEF was significantly smaller than that of ORB and dBRIEF. In the angle range of 20–60°, the SD value of FSD-BRIEF was not as small as mdBRIEF, but smaller than mdBRIEF in the angle range of 70–80°.

Because dBRIEF and mdBRIEF distorted the descriptor template based on the plane perspective model, it could not extract the feature descriptor when θ was 90°, and there was no 90° effective value of the descriptor errors.

It can be observed from the results that, compared with other BRIEF based features, FSD-BRIEF could effectively adapt to the radial distortion of fisheye images and ensure the invariance of descriptors.

### 5.3. Experiment 3: Matching Performance Evaluation in Different Kind of Image Variance

**Dataset:** In order to verify the FoV edge distortion invariance, translation invariance, and scale invariance performances of the proposed FSD-BRIEF in image matching process, a dataset captured by a 210° FoV fisheye camera was made. The intrinsic parameter of the 210° FoV fisheye camera is shown in Table 1. There were three groups of images in this dataset, and each group contained 13 images. In the first group of images, through rotation of the camera, the test image fell on the edge of the camera’s FoV as close as possible, and the test image was distorted by the radial distortion of the fisheye camera to the greatest extent. In the second group of images, by moving and rotating the camera parallel to the test image plane, the test image fell in different positions of the camera FoV. In the third group of images, the camera moved forward and backward greatly relative to the test image, which made the projection of the test image in the fisheye image has a large-scale change.

**Baselines:** In this experiment, five state-of-the-art descriptors, AKAZE, BRISK, ORB, dBRIEF and mdBRIEF, were selected as baselines. For FSD-BRIEF, we used the FAST feature to extract feature points. For BRISK, ORB, and AKAZE, we used the functions provided in OpenCV with default parameter settings. For dBRIEF and mdBRIEF, we used the open source version provided in GitHub.

**Evaluation metrics:** In order to evaluate the matching performance of FSD-BRIEF proposed in this paper, according to [30], we conducted comparison experiments with state-of-the-art descriptors by calculating the PR (recall—“1-precision”) curve of the matching results. Designate $S^{i}$, $S^{j}$ to be a set of feature points detected in the image $I^{i}$ and $I^{j}$ respectively, then the set of ground truth matching points $G^{ij}$ can be given by:

(25) $$G^{ij}=\{\left(p^{i},p^{j}\right)|∥p^{i}-\Pi \left(H^{ij}\Pi^{-1}\left(p^{j}\right)\right)∥<\varepsilon ,p^{i}\in S^{i},p^{j}\in S^{j}\}$$

where $∥\cdot ∥$ refers to Euclidean distance between the $p^{i}$ and the projecting point of $p^{j}$ in image $I^{i}$, $H^{ij}$ is the ground truth homography matrix from image $I^{i}$ to $I^{j}$, which was calculated by manually labeled corresponding points in the image sequence. The distance threshold ε was taken as 3 pixels. To evaluate the matching performance of test features, let $M^{ij}$ be the set of matching feature point pairs gained by the algorithm from the image $I^{i}$ and $I^{j}$, and $M^{ij}$ consisted of correct matches $M_{true}^{ij}$ and incorrect matches $M_{false}^{ij}$. Hence, as shown in Equation (26), the $recall(\varepsilon^{\prime })$ presents the ability of the matching algorithm to find correct matches, and $1-precision(\varepsilon^{\prime })$ indicates the algorithm’s capability of discarding unmatched points.

(26) $$recall\left(\varepsilon^{\prime }\right)=\frac{\sum_{1\leq i<j\leq n}N(M_{true}^{ij}\left(\varepsilon^{\prime }\right))}{\sum_{1\leq i<j\leq n}N(G^{ij})},1-precision\left(\varepsilon^{\prime }\right)=1-\frac{\sum_{1\leq i<j\leq n}N(M_{false}^{ij}\left(\varepsilon^{\prime }\right))}{\sum_{1\leq i<j\leq n}N(M^{ij}\left(\varepsilon^{\prime }\right))}$$

where *n* is the number of images in the image sequence, N(∗) denotes the point pair number of a set, $\varepsilon^{\prime }$ is a descriptor distance threshold that was used to obtain the correct matches whose Euclidean distance between their descriptors is below $\varepsilon^{\prime }$. Each of the two measures yielded a so-called PR cure by increasing the threshold $\varepsilon^{\prime }$ from zero gradually. That PR curve passed at a short distance of the ideal point (0, 1) meant the corresponding test feature was absolutely perfect which could make both the value of precision and recall rate 1. In practice, a good matching performance was achieved when the matching algorithm’s PR curve had the minimum distance to the point (0, 1), the highest recall, and the minimum 1-precision.

**Figure 13:**
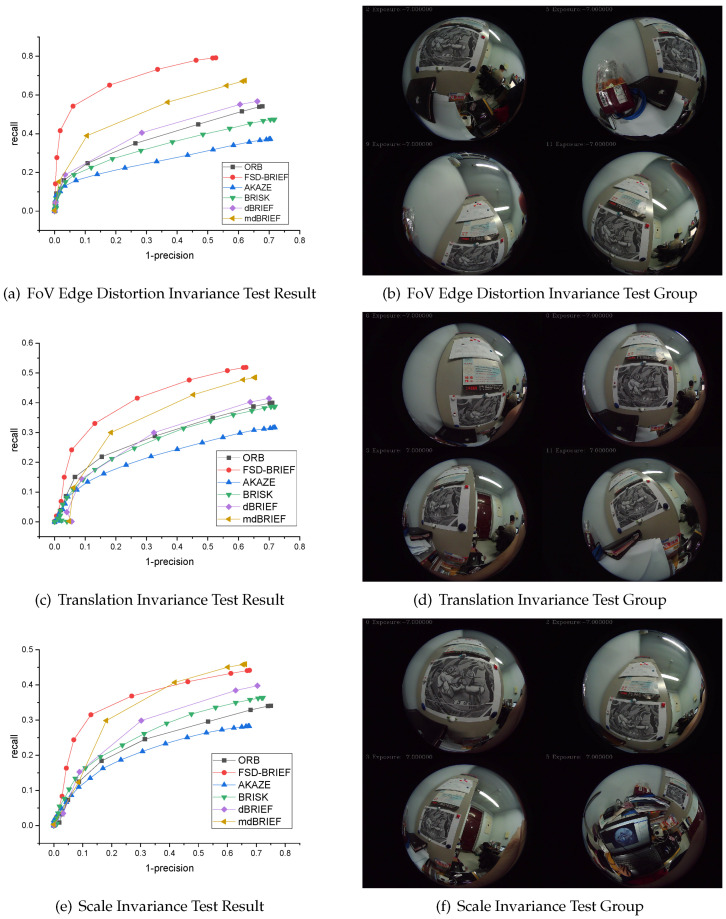
210° FoV camera dataset and corresponding PR curve result.

**Evaluation:** To test the matching performance in this dataset, we used the test features to extract and match features and drew PR curves. For each algorithm in each image, 300 strongest feature points were extracted. The PR curve results are shown in Figure 13.

From Figure 13a,b, the recall value at the end of the PR curve of FSD-BRIEF proposed in this paper was in the range of 0.75–0.8. For other features involved in the comparison, the recall value at the end of the PR curve was in the range of 0.3–0.6. The result showed that, compared with other features, FSD-BRIEF had significant FoV edge distortion invariance in the feature matching process of severely distorted images.

Figure 13c,d shows that the recall value at the end of the PR curve of FSD-BRIEF proposed in this paper was near 0.5. For other features involved in comparison, the recall value at the end of the PR curve was in the range of 0.25–0.5 and below FSD-BRIEF. The result showed that, compared with other features, FSD-BRIEF had better translation invariance in the feature matching process of fisheye images.

In Figure 13e,f, it can be observed that the recall value at the end of the PR curve of FSD-BRIEF proposed in this paper was in the range of 0.4–0.45. For AKAZE, BRISK, ORB, and dBRIEF, the recall value at the end of the PR curve was in the range of 0.25–0.4. The recall value of FSD-BRIEF was higher than mdBRIEF when 1−precision was in the range of 0.05–0.3. The results showed that FSD-BRIEF had better scale invariance in the feature matching process of fisheye images compared with most of the state-of-the-art features.

Using AKAZE, BRISK, ORB, dBRIEF and mdBRIEF as references, experimental results showed that FSD-BRIEF showed comparable performance in FoV edge distortion invariance, translation invariance, scale invariance, and matching performance in fisheye images.

### 5.4. Experiment 4: Matching Performance Evaluation in Different Distortion Images

**Figure 14:**
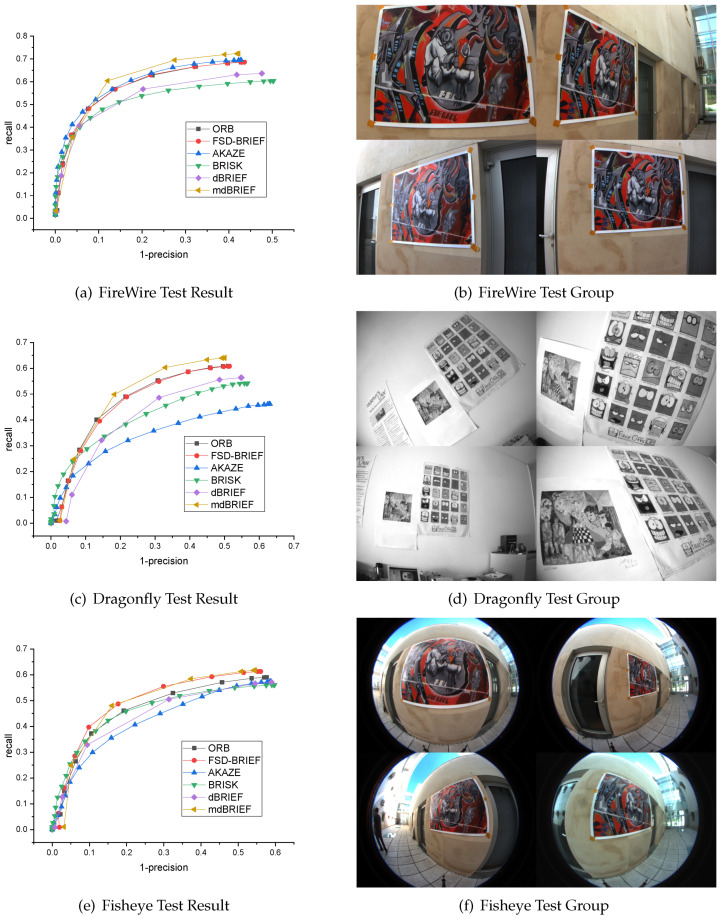
sRD-SIFT dataset and corresponding PR curve result.

**Dataset:** In order to verify the matching performance of FSD-BRIEF under different radial distortion, the sRD-SIFT dataset was used in this experiment. The sRD-SIFT datasets [22] were published with the work of sRD-SIFT. It consisted of three sets of images (FireWire, Dragonfly, and Fisheye), each set containing 13 images and captured by a camera with different radial distortion. The dataset contained significant scaling and rotation changes. Four images selected randomly for each dataset are shown in the right panels of Figure 14.

**Fisheye cameras:** The three sets of images were attached with the image of a checkerboard calibration board for camera calibration. Therefore, we calibrated each camera based on the KB4 fisheye camera model using the chessboard image provided. The calibration results are shown in Table 6.

**Evaluation:** Similar to Experiment 3, to test the matching performance in the three groups of the sRD-SIFT dataset, we also employed the baseline descriptors (ORB, AKAZE, BRISK, dBRIEF and mdBRIEF) to extract and match 300 strongest keypoints for each image, then draw PR curves. The results are shown in Figure 14, where Figure 14a,b shows the results and the image group with the least distortion. Figure 14c,d shows the results and the image group with moderate distortion. Figure 14e,f shows the results of the image group with the most distortion captured by fisheye cameras.

Figure 14a,b shows that the PR curve of FSD-BRIEF almost coincided with that of ORB and AKAZE, and the performance of AKAZE was slightly better. The recall rate at the end of the curve of FSD-BRIEF, ORB, and AKAZE was in the range of 0.65–0.7, which was higher than that of BRISK and dBRIEF. From the result, we can see that the performance of FSD-BRIEF was equivalent to that of ORB in small distorted images.

Figure 14c,d shows that the PR curve of FSD-BRIEF almost coincided with that of ORB, and the recall at the end of the curve was around 0.6, which was higher than that of AKAZE, BRISK, and dBRIEF. From the result, we can see that the performance of FSD-BRIEF was equivalent to that of ORB in moderate distorted images and better than AKAZE, BRISK, and dBRIEF.

In Figure 14e,f, it can be observed that the recall value at the end of the PR curve of FSD-BRIEF was around 0.6, which was higher than that of ORB, AKAZE, BRISK, and dBRIEF, and almost the same as that of mdBRIEF. From the result, we can see that the performance of FSD-BRIEF was almost equivalent to that of mdBRIEF and better than ORB, AKAZE, BRISK, and dBRIEF in the most distorted images.

These experimental results show that the performance of FSD-BRIEF in large distortion image was better than most of the state-of-the-art features involved in the comparison. In small and moderate distorted images, the performance of FSD-BRIEF was similar to that of the ORB feature. That is because that the test image was close to the center of the FoV in this dataset, the radial distortion effect of the test image by the fisheye lens was limited compared with Experiment 3. Therefore, the performance of FSD-BRIEF in this paper on the sRD-SIFT dataset was not as prominent as the 210° FoV camera dataset in Experiment 3.

## 6. Conclusions

In this paper, to tackle the problem of the feature matching performance deterioration due to the impact of fisheye radial distortion, we proposed a novel distorted BRIEF descriptor, named FSD-BRIEF, for fisheye images based on the spherical projection model. First, for reducing the impact of the distortion on gray centroid calculation and the accuracy of feature point direction, we designed a pixel density function and evaluated its performance by comparing the feature point direction error results of the algorithms with and without using the function. The obtained results shown that the pixel density function can promote the precision of the feature point direction calculation. Second, the distortion invariance of the proposed FSD-BRIEF was verified and compared with other BRIEF based descriptors, and the associated results demonstrated that FSD-BRIEF works well for distortion invariance in different positions of fisheye images. In the matching experiments in 210° FoV camera datasets, FSD-BRIEF shown better performance for FoV edge distortion invariance, translation invariance, and scale invariance in large distortion fisheye images. In the sRD-SIFT dataset, the FSD-BRIEF descriptor can significantly improve the matching performance for large distortion images, and meanwhile can still produce excellent results for small distortion images.

## 7. Future Work

It is known that panoramic images have been widely used today. The proposed descriptor can be adapted and potentially applied to panoramic images, with some slight modifications of the camera model and the computation method of the pixel density function, respectively. Moreover, in the future work, we will design a distorted FAST detector based on the spherical perspective model for panoramic images to extract feature points at any position including the two Polar Regions.

## Figures and Tables

**Table 1:** The Intrinsic Parameters of 170° FoV and 210° FoV Camera.

Intrinsic Parameter	170° FoV Camera	210° FoV Camera
$f_{x}$	284.977	257.28
$f_{y}$	284.977	257.28
$c_{x}$	423.039	582.006
$c_{y}$	398.179	419.655
$k_{1}$	−0.00454	−0.0765
$k_{2}$	0.0396	0.00908
$k_{3}$	−0.0363	−0.0117
$k_{4}$	0.00584	0.00373

**Table 2:** The numerical results of direction angle error in 170° FoV camera.

*θ* (°)	Version 1 (Without Compensation)		Version 2 (With Compensation)		Error Reduction (%)	
	*Mean*	*SD*	*Mean*	*SD*	*Mean*	*SD*
10	1.133	0.920	1.084	0.865	−4.306	−5.978
20	1.213	0.827	1.162	0.800	−4.140	−3.360
30	1.034	0.786	0.922	0.703	−10.895	−10.452
40	1.143	0.914	0.948	0.782	−17.111	−14.451
50	1.106	0.905	1.116	0.811	0.895	−10.367
60	1.030	0.796	0.947	0.668	−8.033	−16.065
70	1.756	1.251	0.849	0.656	−51.629	−47.526
80	5.185	3.326	1.342	1.011	−74.123	−69.592

**Table 3:** The numerical results of direction angle error in 210° FoV camera.

*θ* (°)	Version 1 (Without Compensation)		Version 2 (With Compensation)		Error Reduction (%)	
	*Mean*	*SD*	*Mean*	*SD*	*Mean*	*SD*
10	0.697	0.521	0.684	0.504	−1.850	−3.322
20	0.800	0.620	0.781	0.574	−2.425	−7.339
30	1.134	0.840	0.980	0.720	−13.540	−14.277
40	2.052	1.402	1.518	1.080	−25.995	−22.989
50	1.974	1.357	1.218	0.932	−38.280	−31.344
60	1.474	1.163	0.837	0.594	−43.226	−48.942
70	2.085	1.415	0.920	0.717	−55.880	−49.322
80	2.310	1.591	0.899	0.703	−61.068	−55.838
90	2.373	1.605	0.929	0.725	−60.838	−54.859

**Table 4:** The numerical results of Hamming distance error in 170° FoV camera.

*θ* (°)	FSD-BRIEF		ORB		dBRIEF		mdBRIEF	
	*Mean*	*SD*	*Mean*	*SD*	*Mean*	*SD*	*Mean*	*SD*
20	25.100	7.033	25.692	8.005	20.458	6.520	3.458	1.779
30	20.658	6.284	22.767	7.700	18.833	5.974	3.192	1.583
40	21.825	6.994	25.867	8.073	23.850	7.237	4.667	2.413
50	21.300	7.209	30.017	8.798	31.917	9.516	7.083	3.635
60	23.325	7.407	37.050	10.598	44.217	11.882	11.633	5.680
70	26.533	6.904	52.175	12.154	60.742	13.563	19.883	8.170
80	33.850	10.045	87.233	17.572	92.792	15.495	43.083	13.704

**Table 5:** The numerical results of Hamming distance error in 210° FoV camera.

*θ* (°)	FSD-BRIEF		ORB		dBRIEF		mdBRIEF	
	*Mean*	*SD*	*Mean*	*SD*	*Mean*	*SD*	*Mean*	*SD*
20	20.892	5.639	21.158	6.309	17.600	4.467	2.775	1.345
30	22.608	6.125	25.467	7.242	20.000	5.804	3.208	1.460
40	25.767	7.475	30.925	8.592	23.708	7.439	3.792	1.788
50	25.875	7.996	41.442	11.538	31.083	9.718	5.058	1.881
60	28.867	7.978	57.508	15.822	42.558	13.937	8.058	4.101
70	30.317	8.176	70.775	16.628	58.758	14.927	17.892	8.579
80	36.250	10.375	84.217	16.844	95.292	16.522	44.975	14.635
90	45.000	14.170	97.450	21.129	-	-	-	-

**Table 6:** The intrinsic parameters of the cameras in sRD-SIFT datasets.

Intrinsic Parameter	set1(FireWire)	set2(Dragonfly)	set3(Fisheye)
$f_{x}$	539.389	528.626	306.780
$f_{y}$	539.389	528.626	306.780
$c_{x}$	312.103	365.029	634.729
$c_{y}$	233.050	228.558	478.546
$k_{1}$	0.0537	−0.0994	−0.000788
$k_{2}$	0.0871	−0.0205	0.0181
$k_{3}$	0	0.00661	−0.0117
$k_{4}$	0	0.0150	0.00190

**Figure A1:**
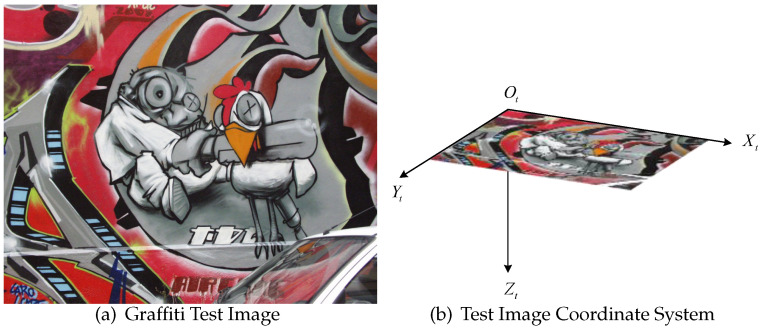
Graffiti Test Image (**a**) and the Test Image Coordinate System (**b**).

**Figure A2:**
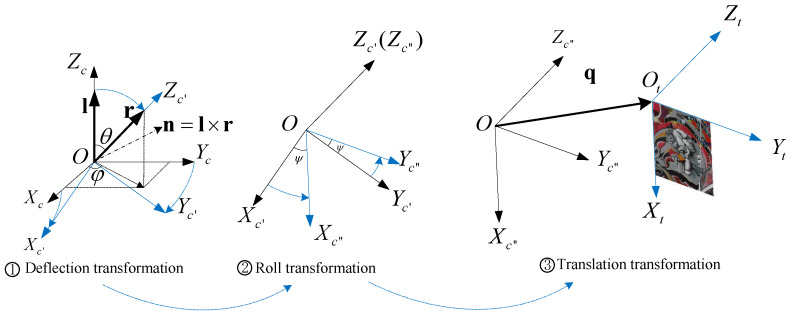
Three step transformation from the camera coordinate system to test image coordinate system.

**Figure A3:**
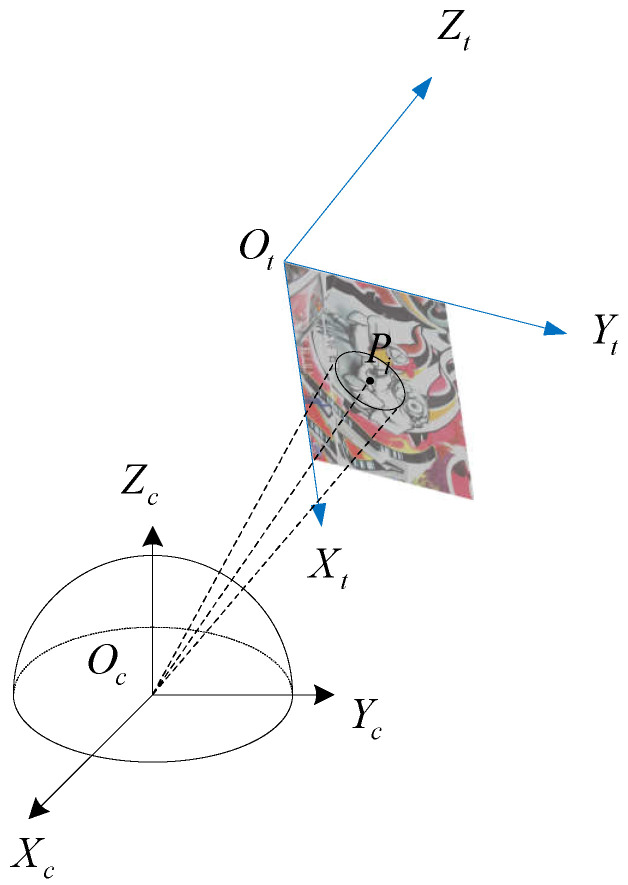
Position relationship between the camera coordinate system and the test image coordinate system.

**Figure A4:**
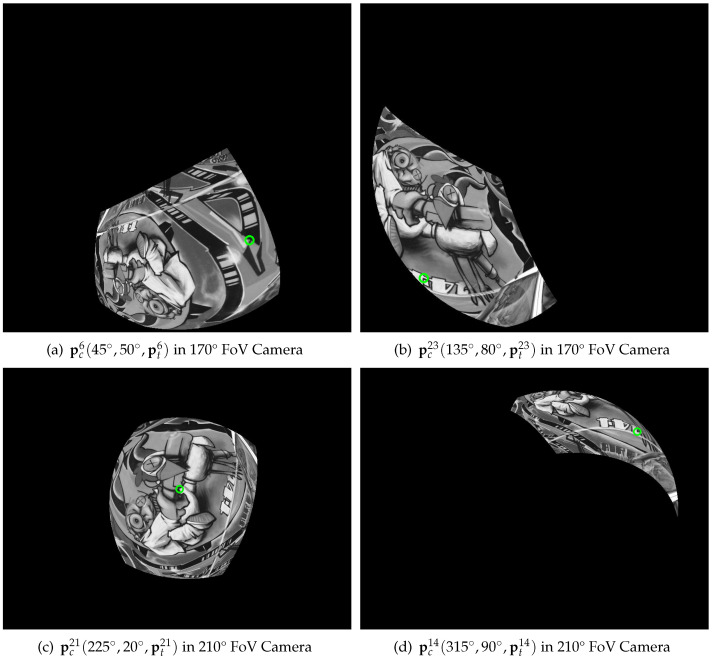
Some examples of virtual dataset test sample. The green circle in sample image indicates the feature point.

## Data Availability

The datasets in Experiment 1-3 are available at https://github.com/Ironeagleufo123/FSD-BRIEF-Dataset (accessed on 6 March 2021). The datasets in Experiment 4 were published with the work of sRD-SIFT.

## Appendix A. Coordinate Transformation for Virtual Dataset Generation

For the test picture shown in Figure A1a, define the test image coordinate system $O_{t}X_{t}Y_{t}Z_{t}$, as shown in Figure A1b. The coordinate origin $O_{t}$ is located in the first pixel in the upper left corner of the test image.

The *X*-axis represents the row of the image pixel, the *Y*-axis represents the column of the image pixel, and the *Z*-axis is determined according to the right-hand rule.

In this paper, the coordinate system transformation process from the camera coordinate system $O_{c}X_{c}Y_{c}Z_{c}$ to the test image coordinate system $0_{t}X_{t}Y_{t}Z_{t}$ is shown in Figure A2, which mainly includes three steps: (1) Deflection transformation, (2) Roll transformation, (3) Translation transformation.

### Appendix A.1. Deflection Transformation

As shown on the left of Figure A2, note that the vector l is the unit vector consistent with the *Z*-axis direction of the camera coordinate system $0_{c}X_{c}Y_{c}Z_{c}$ and the vector r is a 3D unit vector, indicating the position to which the *Z*-axis of the camera coordinate system will turn. The camera coordinate system is rotated around vector l×r according to the right-hand rule, so that the *Z*-axis is consistent with the r vector direction after rotation, and the transition coordinate system $O_{c^{\prime }}X_{c^{\prime }}Y_{c^{\prime }}Z_{c^{\prime }}$ is obtained. The rotation angle is equal to the angle between vector l and r, which is defined as θ.

The unit vector corresponding to the rotation axis l×r is defined as n, and φ is defined as the angle between the projection of the vector r on the $X_{c}O_{c}Y_{c}$ plane and the $O_{c}X_{c}$ axis. The following constraints exist:

(A1) $$\begin{array}{c} \cos\theta =l\cdot r,\\ \sin\theta =∥l\times r∥,\\ n=\frac{l\times r}{∥l\times r∥},\\ n={\left[\begin{array}{ccc} -\sin\varphi & \cos\varphi & 0 \end{array}\right]}^{T} \end{array}$$

According to Rodriguez formula, the coordinate transformation matrix from the transition coordinate system $O_{c^{\prime }}X_{c^{\prime }}Y_{c^{\prime }}Z_{c^{\prime }}$ to the camera coordinate system $O_{c}X_{c}Y_{c}Z_{c}$ is as follows:

(A2) $$R_{cc^{\prime }}=\cos\theta I+\left(1-\cos\theta \right)nn^{T}+\sin\theta n^{\hat{\;}}$$

where the symbol $\hat{\;}$ represents the transformation from three-dimensional column vector to skew-symmetric matrix:

(A3) $$\left(\begin{array}{c} \left[\begin{array}{c} x\\ y\\ z \end{array}\right] \end{array}\right)\hat{\;}=\left[\begin{array}{ccc} 0 & -z & y\\ z & 0 & -x\\ -y & x & 0 \end{array}\right]$$

According to the Equations (A1) and (A2), it can be deduced that:

(A4) $$\begin{array}{cc} R_{cc^{\prime }} & =\left(l\cdot r\right)I+\frac{\left(I\times r\right){\left(l\times r\right)}^{T}}{1+l\cdot r}+\left(l\times r\right)\hat{\;}\\ \; & =\left[\begin{array}{ccc} 1-\frac{\cos^{2}\varphi \sin^{2}\theta }{1+\cos\theta } & -\frac{\sin^{2}\theta \cos2\varphi }{2(1+\cos\theta )} & \cos\varphi \sin\theta \\ -\frac{\sin^{2}\theta \cos2\varphi }{2(1+\cos\theta )} & 1-\frac{\sin^{2}\varphi \cos^{2}\theta }{1+\cos\theta } & \sin\varphi \sin\theta \\ -\cos\varphi \sin\theta & -\sin\varphi \sin\theta & \cos\theta \end{array}\right] \end{array}$$

### Appendix A.2. Roll Transformation

The transition coordinate system $O_{c^{\prime }}X_{c^{\prime }}Y_{c^{\prime }}Z_{c^{\prime }}$ rotates ψ angle around *Z*-axis according to the right-hand rule to obtain the transition coordinate system $O_{c^{{}^{\prime \prime }}}X_{c^{{}^{\prime \prime }}}Y_{c^{{}^{\prime \prime }}}Z_{c^{{}^{\prime \prime }}}$. The transformation matrix between $O_{c^{\prime }}X_{c^{\prime }}Y_{c^{\prime }}Z_{c^{\prime }}$ and $O_{c^{{}^{\prime \prime }}}X_{c^{{}^{\prime \prime }}}Y_{c^{{}^{\prime \prime }}}Z_{c^{{}^{\prime \prime }}}$ is:

(A5) $$R_{c^{\prime }c^{{}^{\prime \prime }}}=\left[\begin{array}{ccc} \cos\psi & -\sin\psi & 0\\ \sin\psi & \cos\psi & 0\\ 0 & 0 & 1 \end{array}\right]$$

### Appendix A.3. Translation Transformation

The transition coordinate system $O_{c^{{}^{\prime \prime }}}X_{c^{{}^{\prime \prime }}}Y_{c^{{}^{\prime \prime }}}Z_{c^{{}^{\prime \prime }}}$ is transformed into the test image coordinate system $0_{t}X_{t}Y_{t}Z_{t}$ through a translation transformation by vector q. The coordinate of vector q in the test image coordinate system is defined as $q_{t}$. Note that the 4×4 relative pose matrix between $O_{c^{{}^{\prime \prime }}}X_{c^{{}^{\prime \prime }}}Y_{c^{{}^{\prime \prime }}}Z_{c^{{}^{\prime \prime }}}$ and $0_{t}X_{t}Y_{t}Z_{t}$ is $T_{c^{{}^{\prime \prime }}t}$, which is expressed as:

(A6) $$T_{c^{{}^{\prime \prime }}t}=\left[\begin{array}{cc} I_{3\times 3} & q_{t}\\ 0^{T} & 1 \end{array}\right]$$

In conclusion, the relative pose matrix $T_{ct}$, between the camera coordinate system $0_{c}X_{c}Y_{c}Z_{c}$ and the test image coordinate system $0_{t}X_{t}Y_{t}Z_{t}$ is as follows:

(A7) $$\begin{array}{cc} T_{ct} & =T_{cc^{\prime }}T_{c^{\prime }c^{{}^{\prime \prime }}}T_{c^{{}^{\prime \prime }}t}\\ \; & =\left[\begin{array}{cc} R_{cc^{\prime }} & 0\\ 0^{T} & 1 \end{array}\right]\left[\begin{array}{cc} R_{c^{\prime }c^{{}^{\prime \prime }}} & 0\\ 0^{T} & 1 \end{array}\right]\left[\begin{array}{cc} I_{3\times 3} & q_{t}\\ 0^{T} & 1 \end{array}\right]\\ \; & =\left[\begin{array}{cc} R_{cc^{\prime }}R_{c^{\prime }c^{{}^{\prime \prime }}} & R_{cc^{\prime }}R_{c^{\prime }c^{{}^{\prime \prime }}}q_{t}\\ 0^{T} & 1 \end{array}\right]\\ \; & =\left[\begin{array}{cc} R_{ct} & R_{ct}q_{t}\\ 0^{T} & 1 \end{array}\right] \end{array}$$

where

(A8) $$R_{ct}=\left[\begin{array}{ccc} 1-\frac{\cos^{2}\varphi \sin^{2}\theta }{1+\cos\theta } & -\frac{\sin^{2}\theta \cos2\varphi }{2(1+\cos\theta )} & \cos\varphi \sin\theta \\ -\frac{\sin^{2}\theta \cos2\varphi }{2(1+\cos\theta )} & 1-\frac{\sin^{2}\varphi \cos^{2}\theta }{1+\cos\theta } & \sin\varphi \sin\theta \\ -\cos\varphi \sin\theta & -\sin\varphi \sin\theta & \cos\theta \end{array}\right]\left[\begin{array}{ccc} \cos\psi & -\sin\psi & 0\\ \sin\psi & \cos\psi & 0\\ 0 & 0 & 1 \end{array}\right]$$

## Appendix B. Virtual Dataset Generation

Several feature points, which are expressed as $\left\{p_{t}^{i}|i=1,2,...,N_{p}\right\}$, are extracted based on FAST feature in the original Graffiti test image, where $p_{t}^{i}={\left[\begin{array}{cc} p_{tx}^{i} & p_{ty}^{i} \end{array}\right]}^{T}$, $N_{p}$ is the number of feature points, and in this experiment, the value of $N_{p}$ is 30. Define that the three-dimensional point corresponding to the feature point $p_{t}^{i}$ in the test image is ${P^{\prime }}^{i}$, and the 3D coordinate of $P^{i}$ in the test image coordinate system is $P_{t}^{i}$, and $P_{t}^{i}={\left[\begin{array}{ccc} p_{tx}^{i} & p_{ty}^{i} & 0 \end{array}\right]}^{T}$. Based on the original ORB centroid calculation method, the gray centroid $c_{t}^{i}$ of each feature point $p_{t}^{i}$ is calculated. Define that the corresponding 3D point of $c_{t}^{i}$ is $C^{i}$, and the 3D coordinate of $C^{i}$ in the test image coordinate system is $C_{t}^{i}$.

In order to ensure that the generated dataset can accurately test the accuracy of the algorithm to calculate the direction of feature points, the dataset generation meets the following conditions:

1. As shown in Figure A3, ensure that the line $\bar{P^{i}O_{c}}$ is perpendicular to the test image plane $X_{t}O_{t}Y_{t}$, that is $\bar{P^{i}O_{c}}⊥X_{t}O_{t}Y_{t}$, so as to ensure that the circular neighborhood used to calculate the gray centroid of the feature point $p_{t}^{i}$ in the test image and the optical center $O_{c}$ of the camera forms a regular cone;
2. Ensure that the length of $\bar{P^{i}O_{c}}$ is equal to the average values of the horizontal and vertical focal length $\frac{f_{x}+f_{y}}{2}$ of the virtual camera, so as to ensure that the circular area used for calculating the gray centroid of feature points in the original test image is approximately the same as that used to calculate the gray centroid of feature points in the fisheye image.

If these conditions are not satisfied, it will lead to the inconsistency between the calculation area of the gray centroid in the original test image and that in the fisheye image, and the calculated gray centroid will have different mathematical meanings, which will lead to the loss of the experimental verification value.

The necessary and sufficient condition for satisfying the above rules is that the vector $q_{t}$ satisfies:

(A9) $$q_{t}\left(p_{t}^{i}\right)={\left[\begin{array}{ccc} -p_{tx}^{i} & -p_{ty}^{i} & \frac{f_{x}+f_{y}}{2} \end{array}\right]}^{T}$$

where $p_{tx}^{i}$ and $p_{ty}^{i}$ are the pixel coordinates of point $P_{t}^{i}$ in the test image, and $f_{x}$ and $f_{y}$ is the horizontal and vertical focal length of the virtual camera. φ and θ determine the projection position of point $p_{t}^{i}$ in the fisheye image. ψ determines the projection position of the gray centroid $C^{i}$ in the fisheye image.

Then, the dataset is generated according to the following method:

1. Within the camera’s FoV, starting from $\theta =10^{{}^{\circ}}$, taking 10° as the interval, the θ angle is uniformly selected, and $N_{\theta }$ values of θ are generated.
2. From 45° to 315°, φ is uniformly taken at 90° intervals, and the number of φ values generated is $N_{\varphi }=4$.
3. When φ is 45° or 225°, the value of ψ is taken as 4θ. When φ is 135° or 315°, the value of ψ is taken as 0.

For each combination of φ, θ and $p_{t}^{i}$, a corresponding fisheye distortion image $I(\varphi ,\theta ,p_{t}^{i})$ is generated, which constitutes a virtual dataset containing $N_{\theta }\times N_{\varphi }\times N_{p}$ fisheye images as follow:

(A10) $$\left\{I\left(\varphi ,\theta ,p_{t}^{i}\right)|\varphi =45{}^{{}^{\circ}},135{}^{{}^{\circ}},225{}^{{}^{\circ}},315{}^{{}^{\circ}};\theta =10{}^{{}^{\circ}},20{}^{{}^{\circ}},\ldots ,80{}^{{}^{\circ}};i=1,2,\ldots ,N_{p};N_{p}=30\right\}$$

The coordinate $P_{c}^{i}$ of point $P^{i}$ and $C_{c}^{i}$ of point $C^{i}$ in the camera coordinate system are calculated by using the coordinate transformation relationship, as shown in the following Equations:

(A11) $$\begin{array}{c} P_{c}^{i}=T_{ct}\left(\varphi ,\theta ,p_{t}^{i}\right)P_{t}^{i}\\ C_{c}^{i}=T_{ct}\left(\varphi ,\theta ,p_{t}^{i}\right)C_{t}^{i} \end{array}$$

The projection position $p_{c}^{i}\left(\varphi ,\theta ,p_{t}^{i}\right)$ of the feature point $p_{t}^{i}$ in the fisheye image is calculated by the following formula:

(A12) $$p_{c}^{i}\left(\varphi ,\theta ,p_{t}^{i}\right)=\Pi \left(P_{c}^{i}\right)$$

According to the relationship shown in the following formula, the ground truth attitude matrix $R{{}_{cb}^{i}}^{*}\left(\varphi ,\theta ,p_{t}^{i}\right)$ corresponding to the feature point $p_{c}^{i}\left(\varphi ,\theta ,p_{t}^{i}\right)$ in the fisheye image is calculated by:

(A13) $$R{{}_{cb}^{i}}^{*}\left(\varphi ,\theta ,p_{t}^{i}\right)=\left[\begin{array}{ccc} \frac{C_{c}^{i}-\frac{C_{c}^{i}\cdot C_{c}^{i}}{P{{}_{c}^{i}}^{2}}P_{c}^{i}}{\left|C_{c}^{i}-\frac{C_{c}^{i}\cdot P_{c}^{i}}{P{{}_{c}^{i}}^{2}}P_{c}^{i}\right|} & \frac{P_{c}^{i}\times C_{c}^{i}}{\left|P_{c}^{i}\times C_{c}^{i}\right|} & P_{c}^{i} \end{array}\right]$$

In this dataset, each virtual fisheye image $I(\varphi ,\theta ,p_{t}^{i})$ uniquely corresponds to a feature point pixel coordinate $p_{c}^{i}\left(\varphi ,\theta ,p_{t}^{i}\right)$ and a ground truth attitude matrix $R{{}_{cb}^{i}}^{*}\left(\varphi ,\theta ,p_{t}^{i}\right)$, which constitutes a feature point test sample. Some examples of test samples are shown in Figure A4.
